# Predatory lizards perceive plant‐derived volatile odorants

**DOI:** 10.1002/ece3.5076

**Published:** 2019-03-19

**Authors:** Jay K. Goldberg, Genevieve Pintel, Stacey L. Weiss, Emília P. Martins

**Affiliations:** ^1^ Department of Biology Indiana University Bloomington Indiana; ^2^ Southwestern Research Station Portal Arizona; ^3^ Department of Biology University of Puget Sound Tacoma Washington; ^4^ School of Life Sciences Arizona State University Tempe Arizona

**Keywords:** foraging strategies, lizards, olfaction, plant indirect defense, tri‐trophic interactions

## Abstract

Many lizards are olfactory foragers and prey upon herbivorous arthropods, yet their responses to common herbivore‐associated plant volatiles remain unknown. As such, their role in mediating plant indirect defenses also remains largely obscured. In this paper, we use a cotton‐swab odor presentation assay to ask whether lizards respond to two arthropod‐associated plant‐derived volatile compounds: 2‐(*E*)‐hexenal and hexanoic acid. We studied the response of two lizard species, *Sceloporus virgatus*and *Aspidoscelis exsanguis*, because they differ substantially in their foraging behavior. We found that the actively foraging *A. exsanguis*responded strongly to hexanoic acid, whereas the ambush foraging *S. virgatus* responded to 2‐(*E*)‐hexenal—an herbivore‐associated plant volatile involved in indirect defense against herbivores. These findings indicate that *S. virgatus* may contribute to plant indirect defense and that a species' response to specific odorants is linked with foraging mode. Future studies can elucidate how lizards use various compounds to locate prey and how these responses impact plant‐herbivore interactions.

## 1 INTRODUCTION

The aroma of plants often mediates their interactions with other organisms in the environment (Dicke & Baldwin, 2010). Such aromas are composed of volatile organic compounds (VOCs) which are small, usually nonpolar compounds with high vapor pressures at room temperature (Harper, 2000). Plant VOCs play crucial roles in mediating above‐ and below‐ground interactions with microbes, other plants, and various animals (Dicke & Baldwin, 2010). Typically, floral VOCs attract pollinators (Kessler, Diezel, Clark, Colquhoun, & Baldwin, 2013), whereas vegetative volatiles serve as “indirect defenses” by attracting predatory animals to the location of herbivorous prey (Kessler & Baldwin, 2001). Indirect defenses have been described as the plant's “cry for help,” and have been the subject of intense scientific inquiry since its initial discovery (Dicke & Baldwin, 2010). Much of the literature is concerned with the role of predatory and parasitoid arthropods in plant indirect defense (Price et al., 1980), although birds can also locate prey using herbivore‐induced plant volatiles (Amo, Jansen, Dam, Dicke, & Visser, 2013). Even though lizards have been shown to aid plant growth via herbivore removal (Spiller & Schoener, 1994; Spiller, Schoener, & Piovia‐Scott, 2016) their responses to plant VOCs and potential role in indirect defense have yet to be thoroughly investigated.

Much of the literature on lizard foraging behavior categorizes species as either actively foraging or ambush foraging (also known as sit‐and‐wait), and active foragers are known to perform greater rates of olfactory behaviors than ambush foragers (Baeckens, Damme, & Cooper, 2017). Lizard olfactory behavior is also known to be linked to a species' diet, with omnivorous lizards using olfactory cues to locate the fruit and flowers that they consume (Cooper, Al‐Johany, Vitt, & Habegger, 2000). Other studies have also shown that omnivorous—but not insectivorous—lizards will respond to plant odors (Cooper, Caldwell, Vitt, Pérez‐Mellado, & Baird, 2002; Cooper & Pérez‐Mellado, 2002). Omnivorous and herbivorous lizards are often direct plant mutualists facilitating seed dispersal and pollination of various plant species (Olesen & Valido, 2003), with at least one plant species expressing a rare trait—colored nectar—that is specifically attractive to lizard pollinators (Minnaar, Köhler, Purchase, & Nicolson, 2013). More recently, an insectivorous lizard has been shown to use the floral volatiles of dead horse arum (*Helicodiceros muscivorus*) to locate their blowfly prey, an apparent side‐effect of this plant attracting pollinators by deceptive mimicry (Pérez‐Cembranos, Pérez‐Mellado, & Cooper, 2018). Despite consistent demonstrations of the importance of olfactory cues in mediating plant‐lizard interactions, no study to our knowledge has addressed the response of lizards to the herbivore‐induced plant odors that are already known to have an indirect defensive function.

We sought to investigate the potential role of insectivorous lizards in plant indirect defenses by determining if they respond to common plant VOCs that are associated with plant indirect defenses. We selected two VOCs known to be involved with the attraction of predators to herbivorous prey: 2‐(*E*)‐hexenal and hexanoic acid. 2‐(*E*)‐hexenal is an herbivore‐induced plant volatile emitted by many plant species (Allmann & Baldwin, 2010; Scala, Allmann, Mirabella, Haring, & Schuurink, 2013), whereas hexanoic acid is a component of insect body odor derived from plant compounds (Weinhold & Baldwin, 2011). These two compounds allowed us to compare a “plant‐emitted” and “insect‐emitted” VOC that are ecologically relevant to our lizard species of interest: the Chihuahuan Spotted Whiptail (*Aspidoscelis exsanguis*) and the Striped Plateau Lizard (*Sceloporus virgatus*), which were selected to allow us to compare an actively foraging species with a sympatric ambush‐foraging species. Furthermore, congeners of both these species are known to consume herbivorous insects and may be locating them via olfactory cues (Stork, Weinhold, & Baldwin, 2011). Given that studies have found that actively foraging lizards perform chemosensory behaviors more frequently than ambush foragers (Baeckens et al., 2017), we predict that actively foraging lizards will show stronger chemosensory responses under all contexts/treatments than ambush‐foraging lizards. We further predict that a compound produced by herbivores will be more salient to lizards (and thus lead to stronger responses) than a chemical produced directly by plants.

To summarize, we ask (a) if lizards are sensitive to a common herbivore‐induced plant volatile, (b) whether a lizard species that uses frequent chemosensory behavior to actively forage for prey is more sensitive to this chemical than an ambush‐foraging species that waits for prey, and (c) if these species are more sensitive to chemicals produced by insect herbivores than our plant volatile of interest.

## 2 METHODS

### 2.1 Study species and chemicals

We quantified the response of two sympatric species of predatory lizard in the Chiricahua Mountains of Arizona, USA to two different volatile organic compounds (VOCs) associated with prey in nature. We chose the Chihuahuan Spotted Whiptail (*A. exsanguis*) and Striped Plateau Lizard (*S. virgatus*) as our study species because these species are representative of the active/ambush‐foraging dichotomy that is often studied in lizards (Baeckens et al., 2017). Lizards of the genus *Aspidoscelis* are predominantly chemically oriented active foragers (Baeckens et al., 2017), whereas *S. virgatus* is a predominantly visually oriented ambush forager (Merker & Nagy, 1984).

We chose two commonly occurring herbivore‐associated VOCs for use in this study: 2‐(*E*)‐hexenal and hexanoic acid. 2‐(*E*)‐hexenal is a green leaf volatile that is a component of the damage‐induced volatile blend of many plants. Two of the most notable are *Nicotiana attenuata* and *Datura wrightii* (Allmann & Baldwin, 2010) which coexist with our lizard species in Arizona. This compound is emitted from plants only while being eaten by an herbivore (Joo et al., 2018). In contrast, hexanoic acid is emitted by herbivores that have fed on acyl sugars present in various desert plants (Weinhold & Baldwin, 2011). Both volatiles are known to be associated with *Manduca sexta* larvae, a known prey item of both whiptail (*Aspidoscelis* spp.) and spiny lizards (*Sceloporus* spp.) in the Mojave Desert (Stork et al., 2011), although they are not exclusively associated with this herbivore or its Solanceous host plants in nature (Scala et al., 2013).

### 2.2 Animal care and housing

**Figure 1:**
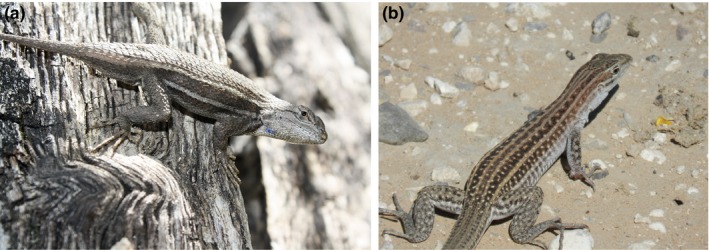
Photos of our study species. (a) *Sceloporus virgatus* (photo credit: Genevieve Pintel) (b) *Aspidoscelis exsanguis* (photo credit: wikimedia commons)

We captured adults of *S. virgatus* (*N* = 43; Figure 1a) and *A. exsanguis* (*N* = 13; Figure 1b) by noose from the area surrounding the Southwestern Research Station (SWRS, Portal, AZ, USA) during May and June 2016. We placed lizards in 37.8 L (10‐gallon) tanks in SWRS' live animal holding facility, with natural substrate, a 60 W heat lamp on a 12:12 light: dark cycle and access to water ad libitum. We offered each lizard 2–4 crickets (*Acheta*sp.) and allowed them to rest for at least 2 days to adjust to captivity before being used in behavioral assays. Some female *S. virgatus* (*N* = 12) were initially housed two per tank and separated by a divider while being used in a separate study, and upon completion were moved to new tanks and cared for as described above.

### 2.3 Chemical cue preparation

We purchased all chemicals from Sigma‐Aldrich (St. Louis, MO, USA) and produced 5 µg/µl solutions of 2‐(*E*)‐hexenal or hexanoic acid in a nonvolatile lanolin matrix. We spread 25 µl of volatile solutions or lanolin control on the tip of a cotton swab immediately prior to each trial. This procedure has been used in previous studies and produces volatile emission rates that are comparable to natural levels (Allmann & Baldwin, 2010, Weinhold and Baldwin 2011).

### 2.4 Chemosensory assay

We presented each of 22 *S. virgatus*males, 21 *S. virgatus*females, and 13 *A. exsanguis* (all parthenogenic females) with three treatments (2‐(*E*)‐hexenal, hexanoic acid, lanolin control) during a single day in May or June 2016. We conducted assays during the period of peak of activity (1000–1700 MST), randomized the order of treatment presentation and waited a minimum of 80 min between consecutive trials for a single animal. During each 5‐min trial, we placed the cotton swab 1 cm from the lizard's nares and counted chemosensory behavior (tongue‐flicks, nose taps, lip licking, and chin rubs) along with their point of contact (at swab, substrate, or air). Tongue‐flicks and nose taps were often difficult to discriminate from one another, and chin rubs and lip licking occurred too infrequently to be analysed independently; thus, we lumped all chemosensory behaviors together for statistical analysis. To minimize bias, the observer was blind to treatment condition while scoring the trial. Some *S. virgatus*(*N*_females_ = 4; *N*_males_ = 4) performed no chemosensory behaviors during any trial. We excluded these nonresponder individuals from statistical analysis. During trials with *S. virgatus* we also recorded the latency to the first chemosensory behavior to contact the swab, but during trials with *A. exsanguis* we instead recorded the latency to the first chemosensory behavior at all.

### 2.5 Statistical analysis

All statistical analyses were conducted using R (R Core Team, 2013). Data collected from *S. virgatus*were not normally distributed and were analysed with nonparametric Friedman's test and Nemenyi's test for post hoc analyses. Data collected from *A. exsanguis* fit the assumptions of parametric tests and were analysed via repeated‐measures *ANOVA* and Tukey's *HSD*.

## 3 RESULTS

### 3.1 *Sceloporus virgatus* response

**Figure 2:**
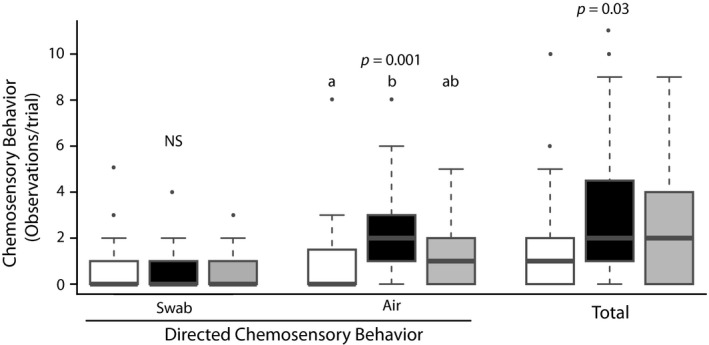
Boxplots summarizing chemosensory behavior performed by *Sceloporus virgatus* during exposures to swabs scented with lanolin (white), 2‐(*E*)‐hexenal (black), or hexanoic acid (gray). Tongue‐flicks were the most frequently observed chemosensory behavior, but these data also include infrequent behaviors such as nose taps, chin rubs, and lip smacking. Letters indicate groups that differ significantly. Air‐directed chemosensory behavior was more frequent during the 2‐(*E*)‐hexenal treatment than during the lanolin treatment. Total chemosensory behaviors also differed between treatments, but post hoc analyses were unable to distinguish pairwise differences. Substrate‐directed chemosensory behaviors were rarely performed by this species and not analysed separately

Chemosensory behaviors contacting the swab did not vary significantly between treatments (*X* ^2^ = 0.67, *p = *0.72, *df* = 2), but those directed at the air did (*X* ^2^ = 13.1, *p = *0.001, *df* = 2; Figure 2). Post hoc analysis indicated a pairwise difference between the air‐directed response to 2‐(*E*)‐hexenal and control lanolin (*p = *0.004, adjusted *α* = 0.01), with a stronger response elicited by 2‐(*E*)‐hexenal. Total chemosensory behavior was also significantly different across all treatments (*X* ^2^ = 6.87, *p = *0.03, *df* = 2; Figure 1), but the low power of nonparametric post hoc analysis could not discern significant pairwise differences. Chemosensory behaviors directed at the substrate occurred infrequently and were not analysed alone but were included in the total number of chemosensory behaviors. Latency to the first tongue‐flick to contact the cue was not found to differ between odorants (*X* ^2^ = 1.56, *p = *0.46, *df* = 2).

### 3.2 *Aspidoscelis exsanguis* response

**Figure 3:**
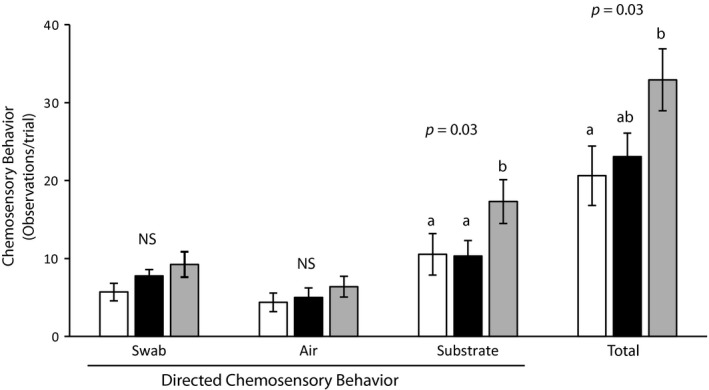
Bar graphs summarizing the mean number of chemosensory acts performed by *Aspidoscelis exsanguis* when exposed to swabs scented with lanolin (white), 2‐(*E*)‐hexenal (black), or hexanoic acid (gray). Tongue‐flicks were the most frequently observed chemosensory behavior, but these data also include infrequent behaviors such as nose taps and chin rubs. Letters indicate groups that differed significantly. Error bars reflect one standard error. Both substrate‐directed and the total count of chemosensory behaviors were more frequent during the hexanoic acid treatment than during other treatment conditions

The number of chemosensory behaviors contacting the cue and air did not differ between treatments (Cue *F*_(2,24)_ = 2.1, *p = *0.15; Air *F*_(2,24)_ = 0.60, *p = *0.56) but those making contact with the substrate did (*F*_(2,24)_ = 4.19, *p = *0.03; Figure 3). Pairwise analysis revealed the number of chemosensory behaviors making contact with the substrate during the hexanoic acid trials to be greater than during the 2‐(*E*)‐hexenal (*p = *0.03) and control lanolin treatments (*p = *0.04), but that 2‐(*E*)‐hexenal did not differ from lanolin (*p = *1.0). The total number of chemosensory behaviors differed across trials (*F*_(2,36)_ = 0.25, *p = *0.03) and pairwise analysis revealed that hexanoic acid elicited a greater response than control lanolin (*p = *0.02) but that no other between treatment differences were present (lanolin:hexenal *p = *0.85; hexenal:hexanoic acid *p = *0.08). The latency to the first tongue‐flick did not differ between treatments (Friedman test: *X* ^2^ = 0.565, *p* = 0.754, *df* = 2).

## 4 DISCUSSION

Foraging mode is known to be phylogenetically linked to baseline rates of chemosensory behaviors with actively foraging species performing these behaviors more frequently (Baeckens et al., 2017). Our results are consistent with this, as *A. exsanguis* performed more chemosensory behaviors than *S. virgatus*. We further found that lizards with different foraging strategies responded to different odorants altogether. This indicates that responses to specific prey‐associated compounds may also be linked to foraging mode, and future comparative studies may be able to tease apart how ecology and evolutionary history dictate the chemical cues used by lizards.

*Aspidoscelis exsanguis* responded to hexanoic acid—a common component of herbivore body and frass odor (Weinhold & Baldwin, 2011)—with elevated rates of chemosensory behaviors that made contact with the substrate. This suggests that they may be searching for nonvolatile cues that they can follow to the location of prey, like the trailing behavior exhibited by snakes (Golan, Radcliffe, Miller, O'Connell, & Chizar, 1982; Kubie & Halpern, 1978). This result was consistent with our prediction that an insect‐derived compound would elicit a stronger response than a plant‐derived compound, however, this only held true for our actively foraging species.

We were surprised to find that *S. virgatus*—an ambush/sit‐and‐wait forager—responded to a plant‐emitted compound. Previous research has found that predatory lizards will not respond to plant‐derived chemical cues (Cooper et al., 2000); however, these studies looked at their response to nonvolatile chemicals and did not investigate responses to plant volatile organic compounds (VOCs). Snakes are known to use prey‐associated chemical cues to select ambush sites (Clark, 2004), and it is possible that *S. virgatus*may use herbivore‐associated plant volatiles to locate more productive ambush sites. Our selected plant VOC – 2‐(*E*)‐hexenal—is a nearly ubiquitous component of the damage‐induced volatile blend produced by plants (Scala et al., 2013), as such it may be a reliable indicator of general increased arthropod density.

Recent studies have shown that insectivorous birds use plant VOCs to locate herbivorous prey (Amo et al., 2013; Mäntylä, Kleier, Kipper, & Hilker, 2017) and that naïve birds lack this response (Amo, Dicke, & Visser, 2016). However, studies with other species of bird, such as the pied flycatcher, have failed to find evidence of plant VOCs being used during foraging (Koski et al., 2015). Our results show that lizards may be behaving in a similar fashion, in which some species use plant VOCs to locate prey while others do not. Although our study did not address whether such behavior is learned or innate, we find it likely that lizards are learning to associate VOCs with the location of prey much like has been shown to occur with birds (Amo et al., 2016).

Although lizards feed upon plant associated arthropods, they may not necessarily benefit plants. A recent study showed that an insectivorous lizard will follow floral volatiles to locate pollinator prey (Pérez‐Cembranos et al., 2018). This plant‐lizard interaction is negative for the plant, and such an outcome can also occur if lizards preferentially feed upon the meso‐predators and parasitoids that benefit plants (Poelman et al., 2012). The potential impact of lizard attraction to a plant is also highly dependent on nonvolatile chemicals, as noxious alkaloids can deter feeding by lizards and other predators of herbivorous insects (Kumar, Pandit, Steppuhn, & Baldwin, 2014; Minnaar et al., 2013).

Lizards have been previously shown to relieve plants of herbivore outbreaks and contribute to trophic cascades that influence plant fitness (Spiller et al., 2016), yet our knowledge of the mechanisms underlying plant‐herbivore‐vertebrate interactions remains sparse. These results serve as a first step toward rectifying this, and present lizards as potential agents of plant indirect defense and selection on plant chemistry.

## CONFLICT OF INTEREST

None declared.

## AUTHOR CONTRIBUTIONS

JKG and EPM conceived the study. JKG, GP and SLW contributed to the planning and execution of the field experiments. JKG analysed data and drafted the manuscript. All authors contributed to and approved the final version of the manuscript.

## ETHICAL STATEMENT

This study complied with ethical standards of animal care and use as outlined by University of Puget Sound permit PS16002 (both species), Arizona Fish and Wildlife permits SP745841 (Sv only) and SP743128 (Ae only). The authors declare no conflict of interest.

## Data Availability

The raw data and R code associated are located at https://doi.org/10.5061/dryad.607cq34.
